# Non-Covalent Interaction on the Self-Healing of Mechanical Properties in Supramolecular Polymers

**DOI:** 10.3390/ijms23136902

**Published:** 2022-06-21

**Authors:** Kwanchai Buaksuntear, Phakamat Limarun, Supitta Suethao, Wirasak Smitthipong

**Affiliations:** 1 Specialized Center of Rubber and Polymer Materials in Agriculture and Industry (RPM), Department of Materials Science, Faculty of Science, Kasetsart University, Chatuchak, Bangkok 10900, Thailand; kwanchai.bu@ku.th (K.B.); phakamat.li@ku.th (P.L.); supitta.sue@gmail.com (S.S.); 2 Office of Research Integration on Target-Based Natural Rubber, National Research Council of Thailand (NRCT), Chatuchak, Bangkok 10900, Thailand

**Keywords:** supramolecular polymer, non-covalent interaction, self-healing product, intrinsic self-healing, self-assembly, molecular recognition, mechanical properties

## Abstract

Supramolecular polymers are widely utilized and applied in self-assembly or self-healing materials, which can be repaired when damaged. Normally, the healing process is classified into two types, including extrinsic and intrinsic self-healable materials. Therefore, the aim of this work is to review the intrinsic self-healing strategy based on supramolecular interaction or non-covalent interaction and molecular recognition to obtain the improvement of mechanical properties. In this review, we introduce the main background of non-covalent interaction, which consists of the metal–ligand coordination, hydrogen bonding, π–π interaction, electrostatic interaction, dipole–dipole interaction, and host–guest interactions, respectively. From the perspective of mechanical properties, these interactions act as transient crosslinking points to both prevent and repair the broken polymer chains. For material utilization in terms of self-healing products, this knowledge can be applied and developed to increase the lifetime of the products, causing rapid healing and reducing accidents and maintenance costs. Therefore, the self-healing materials using supramolecular polymers or non-covalent interaction provides a novel strategy to enhance the mechanical properties of materials causing the extended cycling lifetime of products before replacement with a new one.

## 1. Introduction

Supramolecular chemistry was established and presented by Charles Pedersen, Jean-Marie Lehn, and Donald Cram after their Nobel Prize in Chemistry in 1987s [1,2,3,4,5]. Non-covalent interaction in chemistry was produced to explain the supramolecular chemistry that involved the arrangement of molecules via intermolecular force or non-covalent interactions [6,7,8,9,10,11]. This relatively new part of chemistry deals with self-assembly or molecular assembly that results from the association of two or more chemical species held together by intermolecular interactions [12,13,14,15].

**Figure 1:**
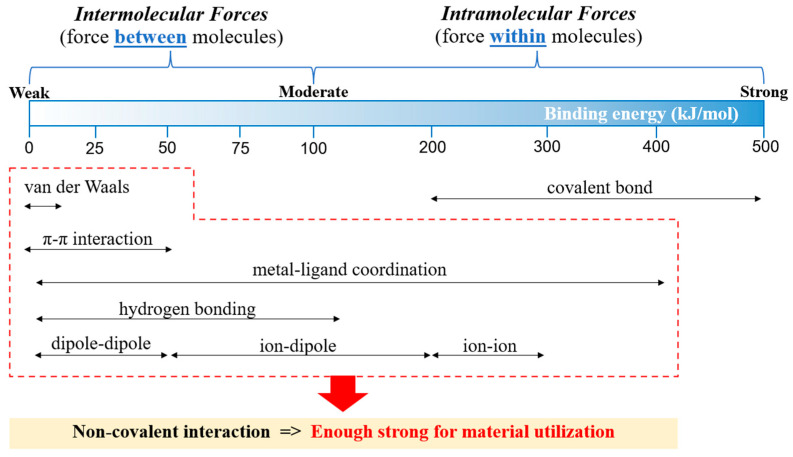
Types of molecular interaction and their binding strength [16]. Adapted from [16], Copyright 2017, with permission from Elsevier.

Generally, the atoms and molecules are linked by the interaction to obtain the strengthened molecules compared to a single atom. The interaction is classified into two types shown in Figure 1: (i) intramolecular forces are covalent interactions, which are forces within molecules such as covalent bonds, and (ii) intermolecular forces are non-covalent interactions, which are forces between molecules, for example, hydrogen bonding, metal–ligand coordination, π–π interaction, van der Waals, ion–ion, ion–dipole, dipole–dipole, etc. Generally, covalent interaction is stronger than the non-covalent interaction. However, non-covalent interactions are strong enough to be maintained and applied for material utilization [16,17,18,19,20].

Therefore, the concept of a combination between covalent bond and non-covalent interaction for molecular recognition is used to design high-performance materials, which can be used as dual crosslinking networks [21,22,23,24]. When the load is applied, the non-covalent interactions are broken before the main backbone of covalent bonds. However, the performance of non-covalent interactions depend on the distance between atoms or molecules. Furthermore, these forces can be reversible and cause a self-healing phenomenon in the materials.

**Figure 2:**
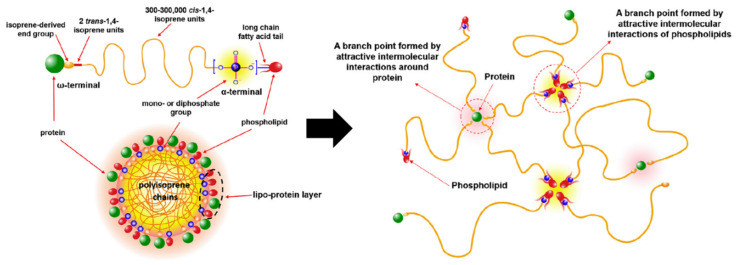
The model of rubber particles and non-covalent interaction in natural rubber molecules [25]. Adapted from [25], Copyright 2018, with permission from Elsevier.

The example of non-covalent interaction in natural rubber was studied by Sriring et al. (2018) [25]. Natural latex can be obtained from the *Hevea brasiliensis* plant [26,27,28,29,30,31]. A rubber particle is suspended in the serum, which consists of non-rubber components such as phospholipids, proteins, carbohydrates, inorganic salts, etc. [32,33,34,35]. The microstructure of rubber exhibits the end chain containing both a ω-terminal and α-terminal. The ω-terminal is normally linked to proteins from the biosynthesis process, whereas the α-terminal is linked to phospholipids. Therefore, the rubber chain with the protein and the phospholipid linkage can be moved and connected together like a network according to the Reptation theory by de Gennes, P.G. (1971) [36,37,38,39,40,41]. This storage hardening phenomenon from the non-covalent interaction between the rubber molecules (as shown in Figure 2) increases the mechanical properties of rubber as a function of time.

**Figure 3:**
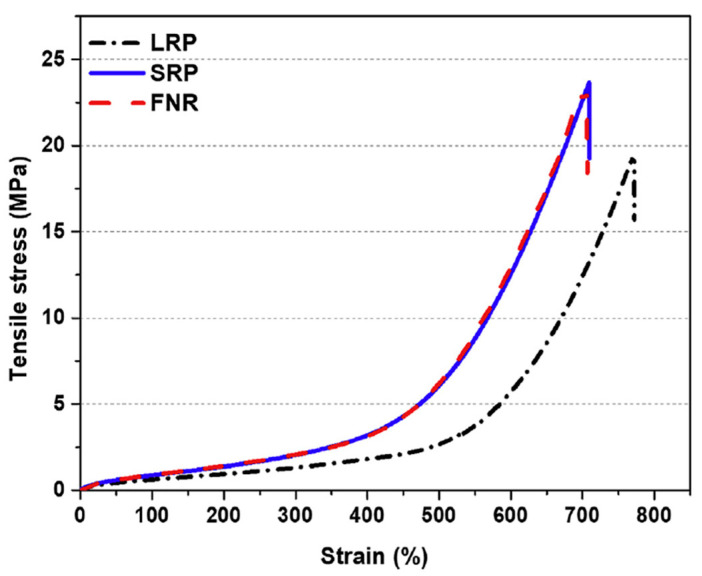
The stress–strain curves of rubber samples with/without non-rubber components [25]. Reprinted from [25], Copyright 2018, with permission from Elsevier.

The results of Sriring et al. (2018) [25] revealed that the mechanical properties of natural rubber (NR) are affected by the non-rubber components in rubber. The tensile strength of the large rubber particles (LRP) sample in Figure 3 was decreased compared to the fresh, natural rubber (FNR) sample due to LRP treated via a centrifugation process, which caused the removal of the non-rubber components and then decreased the physical network representing the non-covalent interaction in the rubber molecules. Furthermore, this phenomenon can be confirmed by Payungwong et al. (2021) [42]. The results found that the crosslink density of deproteinized NR (DPNR) and lipids-removed NR (LRNR) was lower than those of the FNR due to the protein and phospholipid in the molecules being removed. Therefore, the research of Sriring et al. (2018) [25] and Payungwong et al. (2021) [42] can be confirmed and reveal the relationship between the non-rubber components (causing the non-covalent interaction) and mechanical properties of rubber.

**Figure 4:**
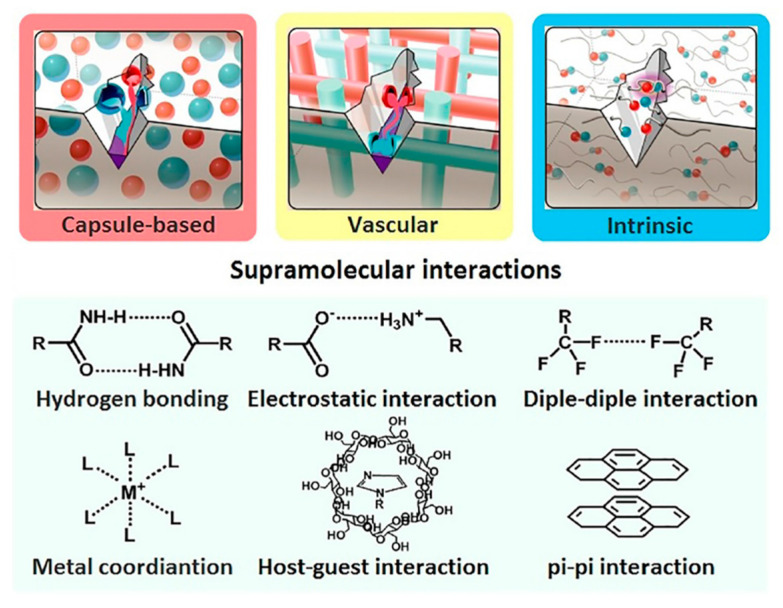
Typical self-healing modes and their chemical structures for self-healing [44]. Adapted from [44], with the permission of AIP Publishing.

Self-healing is a spontaneous healable process of repairing damages in materials. It can be classified into two types such as extrinsic and intrinsic healing based on the previous works from Li and Meng (2015) [43] and Xu et al. (2021) [44]. In the case of extrinsic self-healing materials, the micro- or nano-scale capsule was embedded in the polymer matrix to produce the self-healable process. Nevertheless, the healable efficiency of this system was decreased over time because the healable agents were exhausted during the healing process. In the case of the intrinsic self-healing polymers based on supramolecular or non-covalent interaction, they have advantages including: (i) a fast self-healing process and (ii) reversible bonding in the molecular scale for damage repair. Moreover, supramolecular interactions can produce self-healing within polymer systems such as hydrogen bonding, metal–ligand coordination, electrostatic interaction, host–guest interaction, π–π stacking interaction, dipole–dipole interaction, and van der Waals force (Figure 4). Therefore, the material properties depend on reversible behavior. When the material has a fracture or damage, it is possible to repair or self-heal. So, this material can be used before replacing it with a new one [44,45,46,47,48,49,50,51].

In the case of chemical structures for self-healing, the hydrogen bonding is one of dipole attraction between molecules which is also a non-covalent interaction [5]. Then, the hydrogen bonding can be obtained between a hydrogen atom and a highly electronegative atom, for example, nitrogen, oxygen, or fluorene atoms. Furthermore, it has strengths ranging from 5 kJ/mole to 100 kJ/mole of hydrogen bonding [16]. However, the hydrogen bonds are weak interactions and generally intermolecular bonds, which hold much of soft matter together [52,53,54,55].

In addition, electrostatic interaction is the attractive or repulsive interaction between molecules which have electric charges [5]. These interactions are divided into two types; (i) electrostatic attractions or electrostatic interactions, which occur between molecules that have opposite charges, and (ii) electrostatic repulsions or electrostatic interactions, which occur between molecules that have the same charges [56,57,58,59]. Furthermore, the strengths of electrostatic interaction range from 1 kJ/mole to 25 kJ/mole [16].

Then, the dipole–dipole interactions are intermolecular between two molecules. There are electrostatic interactions between the permanent dipoles of different molecules. The positive charge in polar molecules interacts with the negative charge at the end of another molecule, which exhibits strengths ranging from 10 kJ/mole to 50 kJ/mole [16,60,61,62,63].

Furthermore, metal–ligand coordination is organic–inorganic bonding between metals and ligand atoms related to the Lewis acid base. The Lewis acid within the complexes is an electron acceptor or a central metal ion, which is often a transition metal or inner transition metal. The Lewis base is an electron donor, which is called a ligand. The condition of metal–ligand coordination is that they have one or more electron pairs, which can be donated to the central metal [5]. Therefore, this relates to a donor atom of the ligand with a lone pair of electrons to establish a coordinate interaction with the metal, which widely has strength ranges of 10 kJ/mole to 400 kJ/mole [16,64,65,66,67,68].

Moreover, host–guest interactions are complexes of two molecules or materials through unique structural relationships and non-covalent interactions such as molecular recognition. This interaction is applied in the biorecognition process, for example, via enzyme–inhibitor and antigen–antibody interactions. In addition, π–π interactions occur when the plane of aromatic rings is stacked parallel to one another. This parallel stacking can occur either in a sandwich or displaced stacking arrangement [5,69,70,71].

From a market perspective, self-healing concrete is generally used, which is forecast to have a market value of nearly USD 100 million in 2025 in the USA. These self-healing materials have good performance in repairing crack damage by themselves through embedded capsules. The micro- or nano- capsule releases the healable agents to repair the damaged materials. Then, the market revenue of self-healing materials in the USA from 2020 to 2025 (in USD millions) is shown in Table 1 [72]. Self-healing polymers are also widely applied in many fields, in particular in the applications of soft materials. That is why it is in the top three of market revenue in the United States.

The interdiffusion of rubber molecules plays an important role in self-adhesion between two identical rubbers, especially uncrosslinked or weakly crosslinked rubber [73,74]. This means that the uncrosslinked or weakly crosslinked rubber also represents the self-healable phenomenon. However, the rate of interdiffusion or self-healing of identical soft polymer molecules depends on the structure of the polymer matrix and also the compatibility of chemical substances in the polymer matrix [75]. Interdiffusion is also a function of polymer viscosity; polymers with high viscosity exhibit a long diffusion rate. This interdiffusion phenomenon can also occur with hydrogen bonding interaction in the case of sodium alginate-linked oxidized natural rubber, indicating the rapid self-healing [76].

The self-healing mechanisms relate to viscoelastic properties allowing the molecular mobility to heal the damage and the surface energy creating the contact between two damaged areas. The self-healing of polymers can be succeeded by both covalent and non-covalent networks [77]. From a thermodynamics point of view, the self-healing phenomenon is spontaneous from the favorable Gibbs free energy. The entropy changes come from the conformational chain, while the enthalpy changes come from the chemical reaction in the polymer system [78]. Moreover, the self-healing of a shape memory polymer was modeled based on the constitutive equations to compare the experimental mechanical property with the computational study. The authors found a good agreement between experimental and modelling [79].

**Figure 5:**
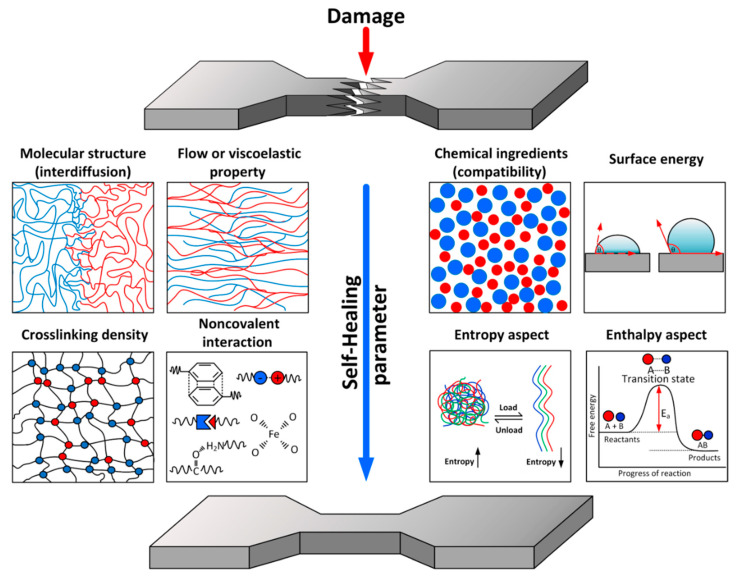
The model of important parameters of the self-healing polymer.

Concerning the self-healing of polymer, the self-healing rate depends on the important parameters shown in Figure 5:

(i) The molecular structure of the polymer (in terms of the rate of interdiffusion of polymer chains);
(ii) The flow or viscoelastic properties of the polymer;
(iii) The chemical ingredients or blended matrix in the polymer composite (in terms of compatible or incompatible materials);
(iv) The surface energy of the polymer;
(v) The crosslinking density (uncrosslinked or weakly crosslinked polymer);
(vi) Non-covalent interaction (metal–ligand coordination, hydrogen bonding, π–π interaction, etc.);
(vii) Entropy aspects (conformational entropy of polymer chains);
(viii) Enthalpy aspects (chemical reaction in the polymer).

Interestingly, self-healing materials can be utilized in advanced applications such as biomimetic, bio-inspired, and smart materials in the robotic field. Tan et al. (2021) [80] presented the roadmap and utilization of self-healing for autonomous robotics, which can potentially apply to conductors, batteries, display screens and lighting, autonomous control, and other electronic devices for humanoid robots, underwater robots, and other biomimetic robots in nano- and micro-scales [80].

Therefore, this review mainly focuses on the very recent technology for the development of self-healing polymers using non-covalent interaction, which has significant potential in material utilization such as metal–ligand coordination, hydrogen bonding, π–π interaction, dipole–dipole interaction, electrostatic interaction, and host–guest interaction.

## 2. Metal–Ligand Coordination of Polymers

**Figure 6:**
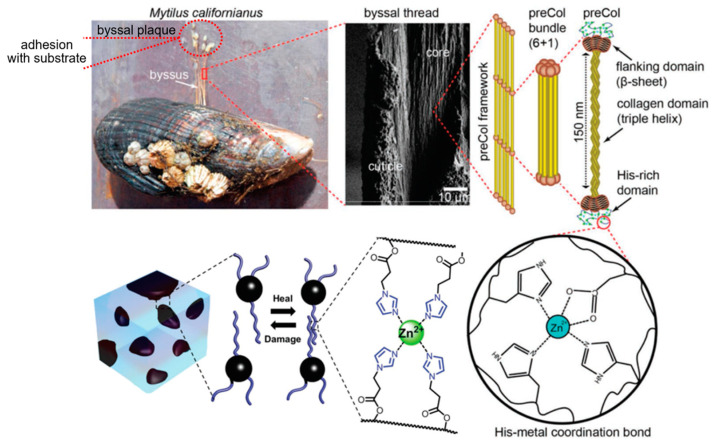
Metal–ligand coordination both in the mussel and between mussel foot protein and substrate [82]. Adapted from [82], with permission from John Wiley and Sons.

The self-healing mechanism may be developed from the metal–ligand coordination in the mussel, which is one of the non-covalent interactions [43]. In nature, the mussel can hold to various substrates such as rocks, metal, wood, and marine organisms using metal–ligand coordination in the byssal thread and byssal plaque surfaces represented in Figure 6. The byssal plaque surface contains the mussel foot protein. The mussel foot protein is an amino acid subsequence with a lot of the catechol group. Therefore, the catechol group can form a coordination bond with substrates to obtain good adhesion. Furthermore, the strength of mussels not only appears in byssal plaque but also in the byssal thread. The metal coordination bonds occurring in the end chain of the collagen in the byssal thread are also shown in this picture. As a result, the byssal thread is able to elongate a lot. The concept of metal–ligand coordination in the mussel using catechol compound is an important key to improving the properties of materials [81,82,83,84].

**Figure 7:**
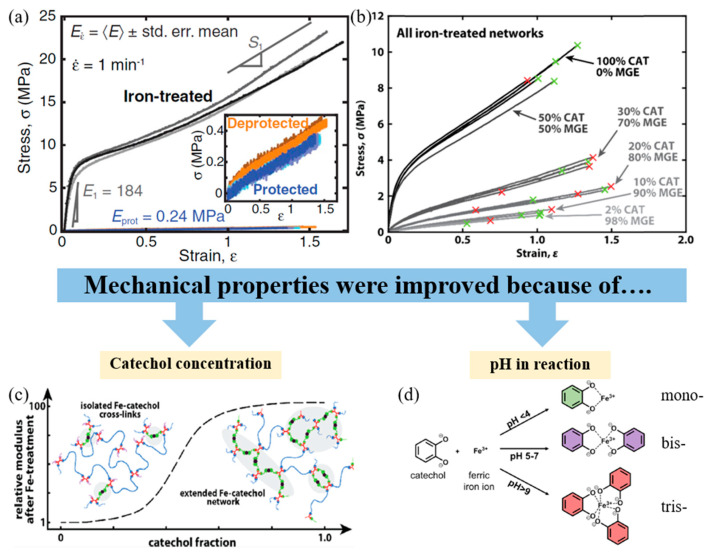
Metal–ligand interaction improves the mechanical properties of coordination complexes depending on the catechol content and pH of the system [85,86,88]. Adapted (**a**) from [85], with permission from AAAS; Adapted (**b**,**c**) with permission from [86]. Copyright 2020 American Chemical Society; Adapted (**d**) from [88], with permission from Springer Nature.

From a research and development point of view, metal–ligand coordination was combined with covalent bonds to produce high-performance materials which exhibit both stiffness and toughness. Filippidi et al. (2017) [85] studied the mussel-inspired iron–catechol complexes in toughening elastomers as poly(ethylene glycol) diglycidyl ethers. It consists of both stiffness and toughness. This phenomenon indicated that stiffness was improved by the coordination between Fe^3+^ and the oxygen atom of catechol. The stiffness of iron-treated materials is higher than that of untreated materials, shown in Figure 7. Furthermore, the effect of the metal–ligand coordination causes the bonds to reform at their original position after unloading. Due to the covalent bond and unbroken metal–ligand coordination, shape memory is preserved to the unloading state. Therefore, the polymer chain can help recovery because of the high interaction between Fe^3+^ and oxygen atoms [85].

The effect of the catechol on the toughening elastomer was confirmed by Cristiani et al. (2020) [86]. The 2-[[3,4-bis[(triethylsilyl)oxy]phenyl]methyl]oxirane or catechol was the same as that used in the research by Filippidi et al. (2017) [85]. In this research, the effect of varying catechol contents on elastomeric properties was investigated. The result revealed that the Young’s modulus of the toughening elastomer is increased with increasing catechol content. Increasing the catechol concentration promotes the formation of the iron–catechol complex site between Fe^3+^ and the oxygen atom of catechol to improve the stiffness, strength, and toughness of the toughening elastomer shown in Figure 7. Furthermore, the tris-formation of the iron–catechol is the best structure to obtain high strength because the metal in crosslinking points can hold the catechol groups more than the mono- and bis-formation depending on the pH of the system [85,86,87].

Metal–ligand coordination is non-covalent interaction between metal and non-metal elements, which has strength ranging from 10 kJ/mole to 400 kJ/mole [16]. A coordination complex can be obtained from metal (Fe^3+^, Fe^2+^, Zn^2+^, Co^2+^, Cu^2+^, Al^3+^, etc.) and ligands, which consists of a lone electron pair such as oxygen in a catechol group or nitrogen in the imidazole ring. So, metal–ligand coordination can possibly be applied in self-healing polymers.

**Figure 8:**
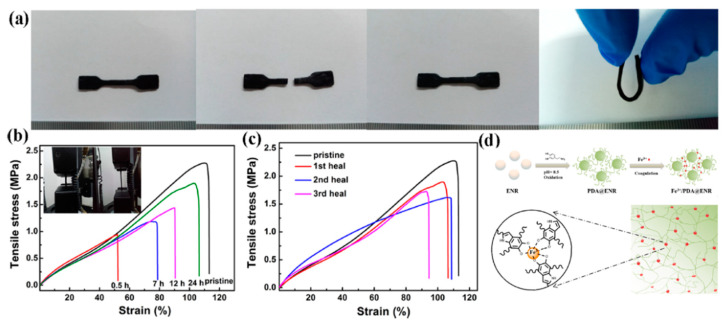
ENR/PDA with Fe^3+^ elastomers and their mechanical properties; (**a**) self-healing testing of ENR/PDA with Fe^3+^ sample, (**b**) the stress–strain curves of ENR samples at various self-healing times, (**c**) the stress–strain curves of ENR samples with various samples of self-heating, and (**d**) the model of ENR/PDA with Fe^3+^ system [89]. Adapted with permission from [89]. Copyright 2017 American Chemical Society.

Self-healing with metal–ligand coordination in the NR was studied by Han et al. (2017) [89]. Epoxidized natural rubber (ENR) is produced by the modification of NR using peracid from formic acid and hydrogen peroxide to obtain the epoxide groups in the NR molecules. In this research, the ENR was reacted with dopamine, which is one of the catechol compounds, to obtain the grafting of dopamine (PDA) onto ENR molecules using self-polymerization. Then, the ENR/PDA was crosslinked by Fe^3+^ to form reversible Fe^3+^–catechol coordination interactions shown in Figure 8. The results revealed that the ENR/PDA with Fe^3+^ sample is cut into two pieces and reconnected, which can be bent again without fracture. Therefore, the self-healing process was obtained using metal–ligand coordination. Furthermore, the healing performance in terms of the mechanical properties was increased with the increase in healing time [89].

**Figure 9:**
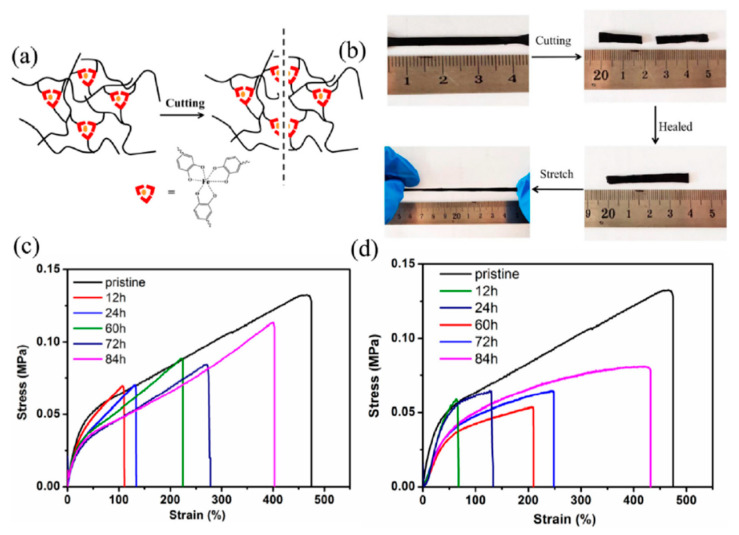
Self-healing of silicone elastomer via amino groups and mechanical properties; (**a**) network structure of silicone elastomer, (**b**) self-healing testing in silicone elastomer, (**c**,**d**) stress–strain curves of healed samples at various healing times for 120 °C and underwater (pH = 9) [90]. Reprinted from [90], Copyright 2020, with permission from Elsevier.

Self-healing with metal–ligand coordination was studied in synthetic rubber by Lia et al. (2020) [90]. In this research, methyl vinyl silicone rubber (MVQ) was dissolved in a tetrahydrofuran solvent and reacted with DOPA and FeCl_3_·6H_2_O. The mixture was poured into a polytetrafluoroethylene (PTFE) Petri dish. Then, it was dried at 60 °C for 24 h under a vacuum to obtain a silicone elastomer with DOPA and Fe^3+^. The results in Figure 9 reveal that the silicone elastomer with DOPA and Fe^3+^ could be healed at both high temperatures and in underwater (pH = 9) conditions [90].

**Figure 10:**
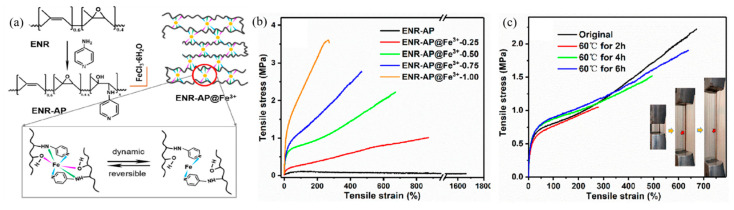
Self-healing of ENR elastomers via amino groups and their mechanical properties; (**a**) the mechanism of ENR elastomer with metals and pyridine ligands, (**b**,**c**) the stress–strain curves of ENR elastomer with various Fe^3+^ content and various self-healing times at 60 °C [91]. Adapted from [91], Copyright 2021, with permission from Elsevier.

Furthermore, metal–ligand coordination can be obtained from the reaction between pyridine ligands with Fe^3+^, which was discovered by Cao et al. (2021) [91]. The ENR with pyridine ligand was prepared by the ring-opening reaction of epoxy groups on ENR molecules and interacted with amino groups to obtain the grafting of pyridine ligands onto ENR molecules. The coordination between pyridine ligands with Fe^3+^ is presented in Figure 10a. The DSC thermograms revealed that the glass transition temperature of ENR-AP was increased with increasing Fe^3+^ contents due to the coordination between pyridine ligands with Fe^3+^. This interaction obstructed the chain mobility, which confirmed the metal–ligand coordination in the molecules and increased the mechanical properties, as shown in Figure 10b. Moreover, the healing performance in terms of the tensile strength and elongation at break in Figure 10c were increased by increasing the healing time, as in Han et al. (2017) [89,91].

The characteristics of metal–ligand coordination can be investigated by FT-IR, Raman, and UV/VIS spectroscopy, which are summarized in Table 2.

## 3. Hydrogen Bonding of Polymers

In the case of hydrogen bonding, this bond is a strong intermolecular interaction between hydrogen atoms and high electronegativity atoms such as nitrogen, oxygen, or fluorene atoms. H-bonding is also widely used for self-healing applications to repair damage and improve the mechanical properties of polymers [43].

**Figure 11:**
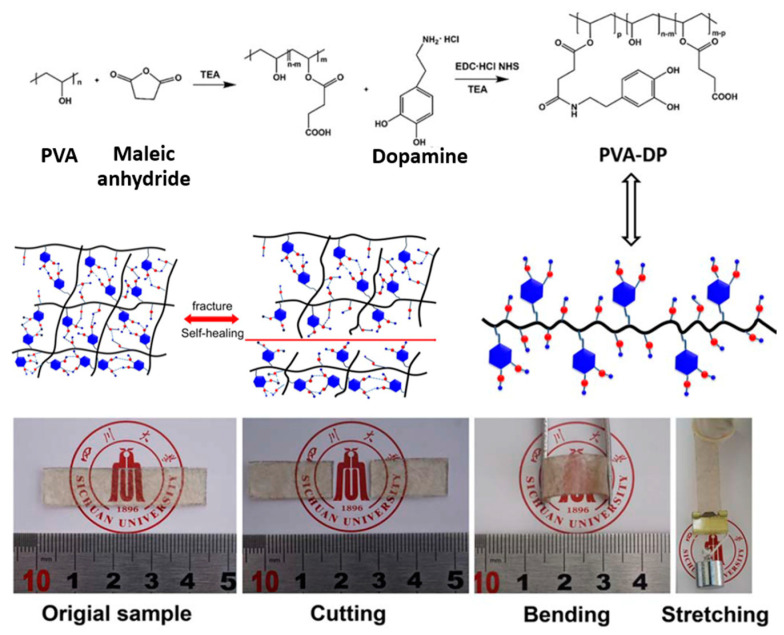
The network formation and self-healing process of the modified poly(vinyl alcohol) elastomer [95]. Adapted from [95], with permission from John Wiley and Sons.

Li and Xia (2017) [95] studied the self-healing of modified poly(vinyl alcohol) or PVA using hydrogen bonding. The PVA reacted with succinic anhydride using grafting modification to obtain the modified PVA. Then, dopamine hydrochloride was reacted with modified PVA via carboxylation, as shown in Figure 11, to produce the dopamine functionalized PVA, which contains the catechol in the PVA chains [95]. The sample can be bent and stretched again after self-healing via hydrogen bonding.

**Figure 12:**
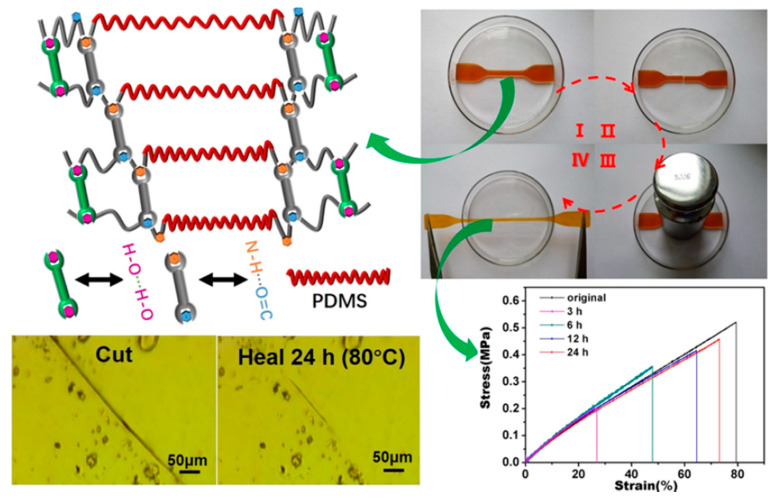
Hydrogen bonding network with silicone rubber (PDMS) represents the self-healing properties [96]. Reprinted with permission from [96]. Copyright 2019 American Chemical Society.

Liu et al. (2019) [96] presented another type of self-healing of silicone rubber, which represented the hydrogen bonding of silicone rubber (HBSR) using multiple hydrogen bonds of α, ω-aminopropyl poly(dimethylsiloxane), and ethylene carbonate based on the non-isocyanate reaction. The results (Figure 12) revealed that multiple hydrogen bonding is obtained between the carbonyl and imino groups as well as the generated hydroxyl groups. In terms of the mechanical properties, tensile strength and elongation at the break of the healable sample at 24 h can be reached at almost 90% compared to those of the original sample. These mechanical properties of the original HBSR sample are equal to or even better than the conventional vulcanized silicone rubber. Moreover, multiple hydrogen bonding also led to silicone rubber exhibiting thermal-induced self-healing properties at 80 °C. Therefore, this research provided an alternative method to develop self-healing silicone rubber with multiple hydrogen bonding [96].

**Figure 13:**
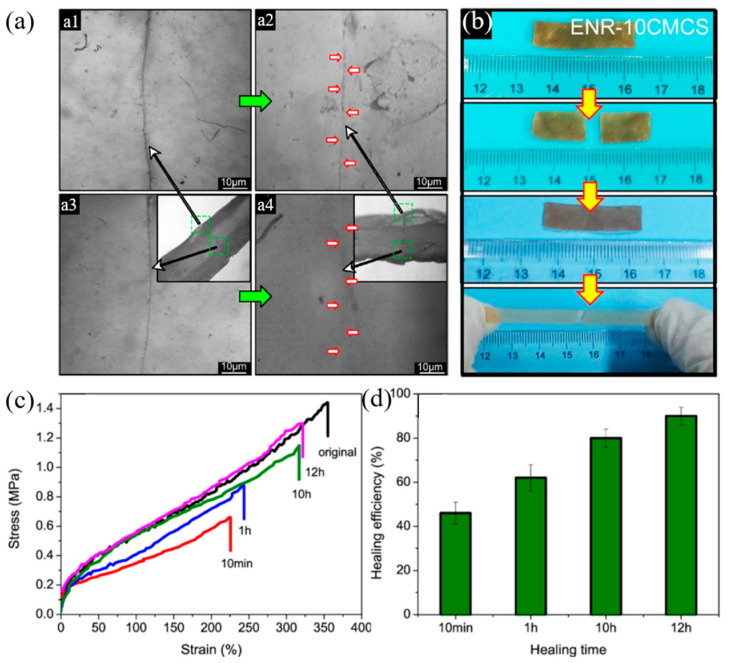
The self-healing microscope and photograph (**a**,**b**) ((**a1**) the cutting line at the surface before self-healing; (**a2**) the cutting line at the surface after self-healing; (**a3**) interior of the cut position before self-healing; (**a4**) interior of the cut position after self-healin for 12 h at room temperature), stress–strain curves at various healing times (**c**) and healing efficiencies (**d**) of epoxidized natural rubber with carboxymethyl chitosan composites [97]. Adapted with permission from [97]. Copyright 2019 American Chemical Society.

Xu et al. (2019) [97] reported non-covalent networks by incorporating carboxymethyl chitosan (CMCS) into epoxidized natural rubber (ENR) to obtain hydrogen bonding using a solution-mixing method. The results revealed that hydrogen bonding is formed by multiple hydrophilic groups of CMCS with ENR chains to obtain the multi-linkages as the supramolecular networks in the molecules. In addition, hydrogen bonding can improve the healing system and mechanical properties of the ENR with CMCS composites (Figure 13). They found that the ENR with 5 and 10 wt.% CMCS improve tensile strengths of 1.40 and 1.92 MPa, respectively. Then, these materials represented self-healing efficiency of almost 90% at room temperature and healable time at 12 h. In the case of CMCS content over 10 wt.%, although the mechanical properties still increased, the self-healing efficiency degraded significantly because of the agglomeration of CMCS filler. Furthermore, hydrogen bonding produced supramolecular networks to improve the recycling capacity of ENR with CMCS composites [97].

**Figure 14:**
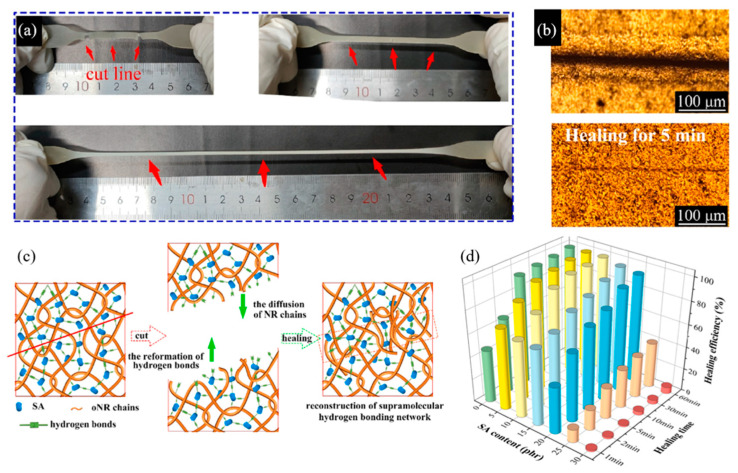
The self-healing photograph and microscope (**a**,**b**), self-healing mechanism (**c**), and healing efficiency (**d**) of SA crosslinked oNR supramolecular networks [76]. Adapted from [76], Copyright 2021, with permission from Elsevier.

Shen et al. (2021) [76] studied rubber networks based on supramolecular hydrogen-bonding networks of oxidized natural rubber (oNR) crosslinked with sodium alginate (SA) to obtain a rapidly self-healing composite film. The result showed that the oNR composite with 20 phr SA exhibited mechanical properties improving in terms of tensile strength to 6.5 MPa. Moreover, the photographs and optical microscopy images of the self-healing process of the oNR/SA film are presented in Figure 14. This result indicated the self-healing efficiency could reach 60% after 2 min of healing time and reach 80% after 10 min of healing time at room temperature [76].

**Figure 15:**
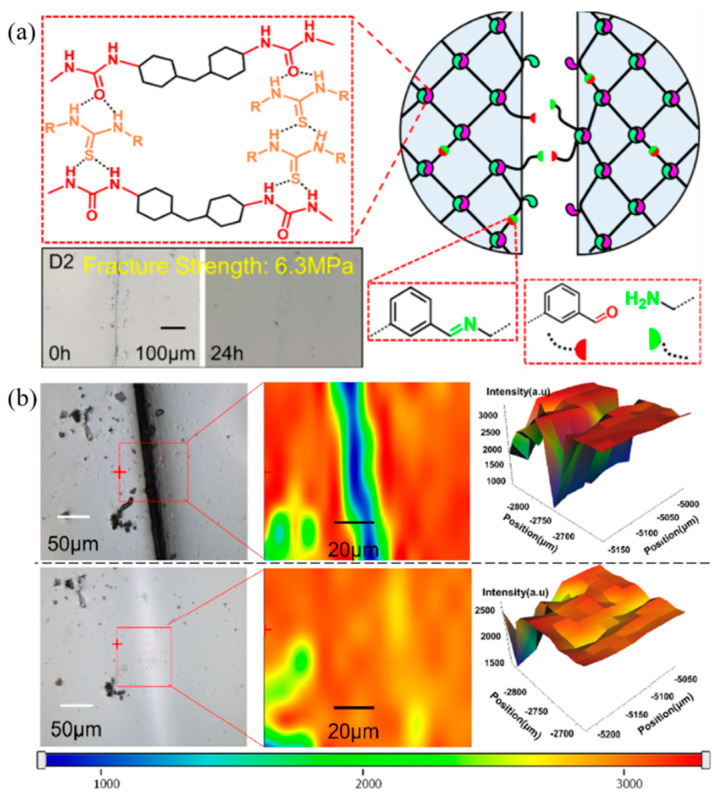
The self-healing model and microscope of silicone elastomers with hydrogen bonding and covalent bonds; (**a**) the model of broken and healing mechanism; and (**b**) the mapping mode of Raman spectra in the healing process [98]. Adapted with permission from [98]. Copyright 2022 American Chemical Society.

Furthermore, the synergistic effect of self-healing in the silicone elastomer based on dynamic covalent bonds and multiple hydrogen bonding was studied by Chen et al. (2022) [98]. The network structure in Figure 15 present that the multiple bonds are obtained by adding thiourea into the polyurea network. The dynamic covalent bonds of imine groups provide materials with a strong link to the damaged surface. The results found that this method can be improved for self-healing efficiency without degrading the mechanical properties of the elastomer. Furthermore, the Raman spectra using mapping mode presented self-healing behavior and healable efficiency of almost 79% for 1 h and 94% for 6 h of healing time. Therefore, the optimized self-healing process presented interpenetration diffusion of the rubber chain and rearrangement of network join between the two interfaces to obtain a rapidly self-healing and tough material [98].

The characteristics of H-bonding in self-healing polymers were investigated using FT-IR and ^1^H-NMR, which are summarized in Table 3.

## 4. π–π Interaction of Polymers

**Figure 16:**
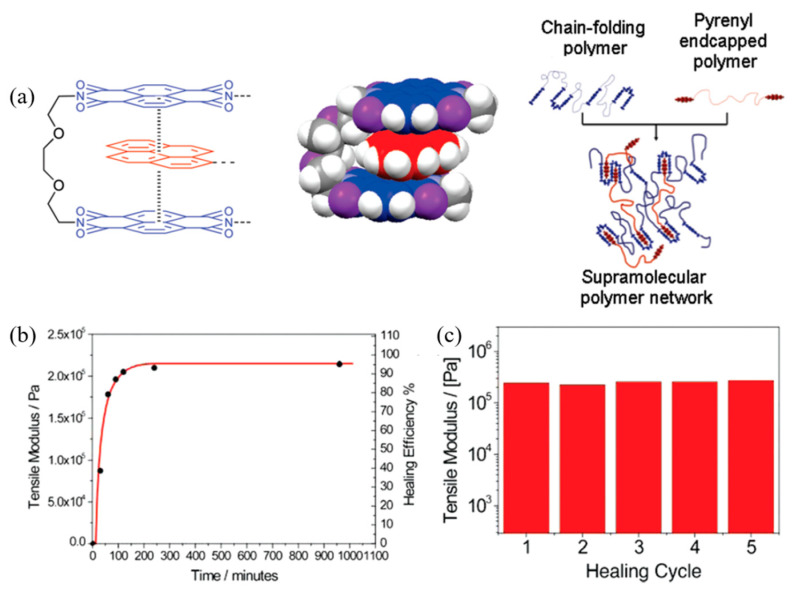
The supramolecular polymer network with π–π interaction; (**a**) π–π interaction mechanism of pyrenyl polymer and aromatic ring in chain-folding polymer, (**b**) tensile modulus and healing efficiency as function of time, and (**c**) tensile modulus under five breaks-heal cycles [102]. Adapted with permission from [102]. Copyright 2010 American Chemical Society.

The π–π stacking interactions are non-covalent interactions between aromatic compounds containing π orbitals which can be arranged in two types; (i) face-to-face stacking and (ii) edge-to-face stacking [43,99]. It can be used in many applications such as self-assembly, self-healing materials, molecular receptors, controlled drug release, fabrication and sensors, composites, and function materials with supramolecules to produce advanced properties [100]. Burattini et al. (2009) [101] studied the novel supramolecular polymer system for self-repairing by π–π stacking interactions. The terminal pyrenyl groups of polyamide were inserted into the chains of a polyimide through complementary π–π stacking. The result found that the new material exhibited an improved ability to flow and was self-healable compared to conventional thermoplastics. The healable process was very fast at 80 °C depending on the healing time [101]. Furthermore, the polymer blend based on aromatic π–π stacking and hydrogen bonding interactions was investigated by Burattini et al. (2010) [102]. The results in Figure 16 revealed the tensile modulus and healing efficiency of the damaged material as a function of healing time, which exhibits a maximum healing efficiency of 95% after a healing time of approximately 240 min [102]. Therefore, these results indicated that π–π stacking could be applied for self-healing applications.

**Figure 17:**
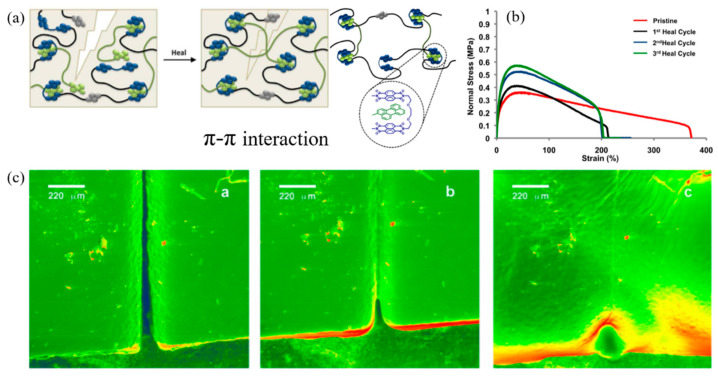
The self-healing model in perylene polymer and chain-folding polydiimide; (**a**) healing mechanism in polymer chain with π–π interaction, (**b**) stress–strain curves of pristine and samples with various healing cycles, and (**c**) ESEM images of healing polymer with π–π interaction (a: 25 °C, b: 75 °C, c: 125 °C) [103]. Adapted from [103], Copyright 2015, with permission from Elsevier.

Furthermore, the research of Hart et al. (2015) [103] confirmed the self-healing behaviors via π–π interaction. Figure 17 show the mechanism of π–π interaction in the molecules. The results revealed that the healing process is rapidly and fully healed at 75 °C for 40 min or 125 °C for 14 min, as shown in environmental scanning electron microscopy (ESEM) images. Moreover, the tensile modulus of 10 MPa exhibited 100% recovery over three break–heal cycles. Therefore, this research demonstrates the ability of the new perylene-based non-covalent interaction and the ability to tailor π–π interaction to promote self-healing polymers.

The characteristics of π–π interaction for self-healing processes were investigated using FT-IR, and UV/VIS spectroscopy, which is summarized in Table 4.

## 5. Electrostatic Interaction of Polymers

Electrostatic interaction is one of van der Waals interactions relating to the attractive or repulsive interaction between atoms, which consists of electric charges. It can be applied in self-healing polymers due to electric charges between atoms and to obtain matrix repairing.

**Figure 18:**
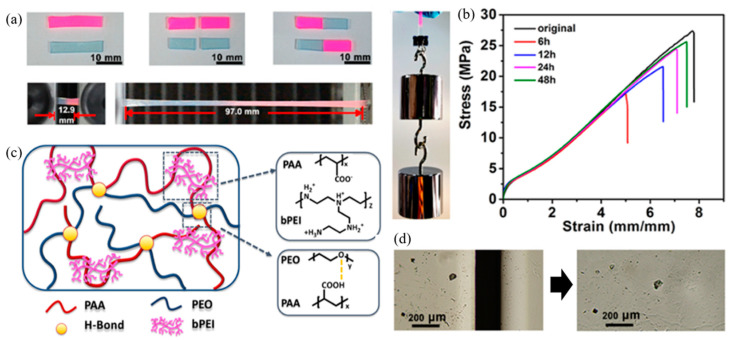
The self-healing of toughening polymers using dual dynamic crosslinked complexes; (**a**) self-healing samples were stretched under tension and lifted a weight after the healing process, (**b**) stress–strain curves of pristine and healed samples with various healing times for 6, 12, 24, and 48 h at room temperature, (**c**) network structure of complexes from electrostatic interaction and hydrogen bonding, and (**d**) optical images of damaged and healed sample after healing time for 48 h at room temperature [106]. Adapted with permission from [106]. Copyright 2019 American Chemical Society.

Guo et al. (2019) [106] presented the self-healing of tough polymers from the polymeric complexes between branched poly(ethylenimine) or bPEI, poly(acrylic acid) or PAA, and poly(ethylene oxide) or PEO using dual dynamic crosslinked polymers. The dual dynamic interactions consist of the hydrogen bonding between PAA and PEO and electrostatic interactions between PAA and bPEI, which are presented in Figure 18. The results revealed that the maximum stress and elongation at break of the storage sample for 48 h reach 25.7 MPa and 750%, respectively. Furthermore, this result indicated that the mechanical properties are completely returned to their original stage after the self-healing process. The self-healing property exhibited a higher, which related to the strong dynamic electrostatic interactions and hydrogen bonding [106].

**Figure 19:**
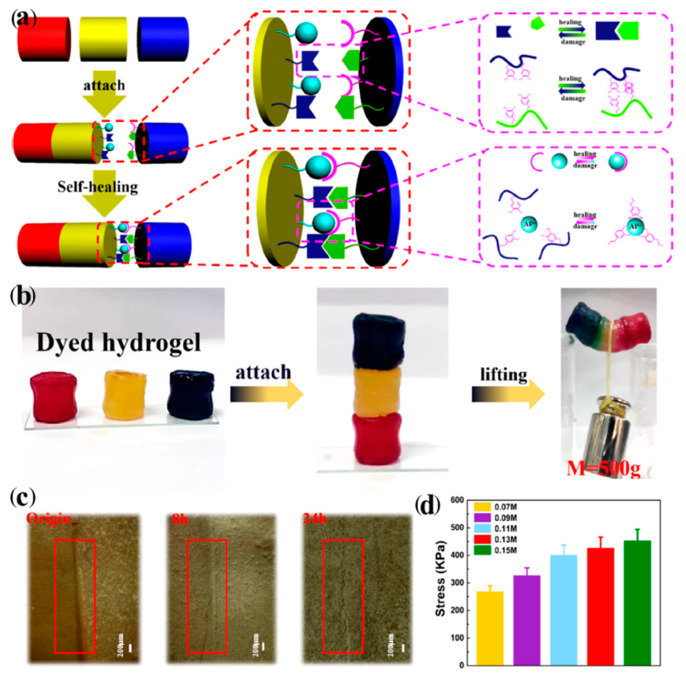
The self-healing in the mussel-inspired hydrogel system; (**a**) mechanism of self-healing process, (**b**) healing process and its experiment by lifting a weight, (**c**) ultra depth micrographs of damaged and healed samples, and (**d**) compressive modulus of hydrogels at various ion concentrations [107]. Adapted with permission from [107]. Copyright 2021 American Chemical Society.

The mussel-inspired antibacterial hydrogel using electrostatic interactions, coordination bonds, and hydrogen bonds for self-healing was studied by Deng et al. (2021) [107]. The results in Figure 19 indicate the self-healing mechanisms. The Al^3+^ on the fracture interface still reactivated to the alginate-dopamine or Alg-DA chains using a coordination interaction, while Al^3+^ was diffused near the fracture surface to help the mobility of the Alg-DA chains. This promoted the rearrangement of coordination and electrostatic interactions. The ultra depth microscope was used to observe the damage–heal process that the sample was self-healable after 8 h and almost restored to the original stage after 24 h. Furthermore, the compressive stress increased with the ion concentration due to the strong interaction [107].

Furthermore, Su et al. (2021) [108] studied the conductive self-healing hydrogel in terms of network structure, healing, and mechanical properties. The results revealed the multiple supramolecular in the molecules as electrostatic interaction, hydrogen bonding, and covalent bonds. The stress–strain curves of hydrogel samples indicated the healed samples could recover their mechanical properties with the increase in healing time. After healing for 12, 24, 48, and 72 h, the tensile strength and strain at 72 h were recovered to the nearly original stage. So, the mechanical properties of hydrophobic association poly(acrylic acid)/polyaniline (PAAN) hydrogel were improved without requiring the self-healing efficiency of the hydrophobic association poly(acrylic acid) or HAPAA hydrogel matrix, compromising the balance between mechanical and self-healing properties.

The characteristics of electrostatic interaction for self-healing in materials were investigated using FT-IR, UV/VIS spectroscopy, and ^1^H-NMR, which are summarized in Table 5.

## 6. Dipole–Dipole Interaction of Polymers

Dipole–dipole interactions are also non-covalent interactions, which are weaker than those of other interactions. They can be obtained from the interaction of two dipolar molecules. The role of dipole–dipole interactions in the self-healing process is the chain movement of polar molecules to produce polymer matrix repairing.

**Figure 20:**
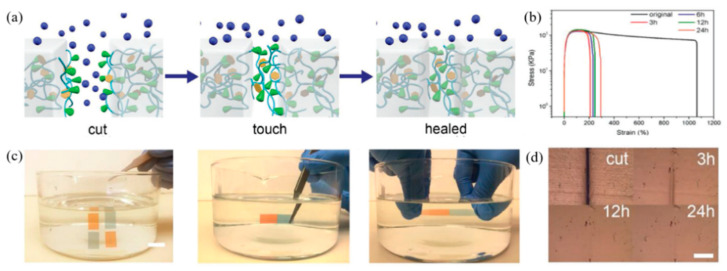
The self-healing of fluorinated elastomer using dipole–dipole interaction; (**a**) healing mechanism underwater, (**b**) stress–strain curves of healed samples with various healing times at room temperature, (**c**) healing demonstration underwater, and (**d**) optical images of damaged and healed samples [109]. Adapted from [109], with permission from John Wiley and Sons.

Cao et al. (2018) [109] studied self-healing elastomers using dipole–dipole interactions and demonstrated a self-healing process underwater. In this research, the elastomer was prepared by mixing poly(vinylidene fluoride-co-hexafluoropropylene) (PVDF-HFP) with various plasticizers, including succinonitrile (SN), dioctyl phthalate (DOP), and dibutyl phthalate (DBP), respectively. The results found that the elongation at the break of the PVDF-HFP elastomer with DBP was higher than those of SN and DOP. So, PVDF-HFP/DEP was used to study the self-healing underwater due to the high hydrophobic behavior of fluorinated elastomer and DBP. The healing mechanism at various healing times was observed by a microscope, as shown in Figure 20. After the healing test, the elastomer can be stretched to 200% strain, indicating the self-healing of the elastomer at a healing time of 3 h. Furthermore, the microscope shows fully healing in the range of 12–24 h, which disappears the crack of the damaging surface.

**Figure 21:**
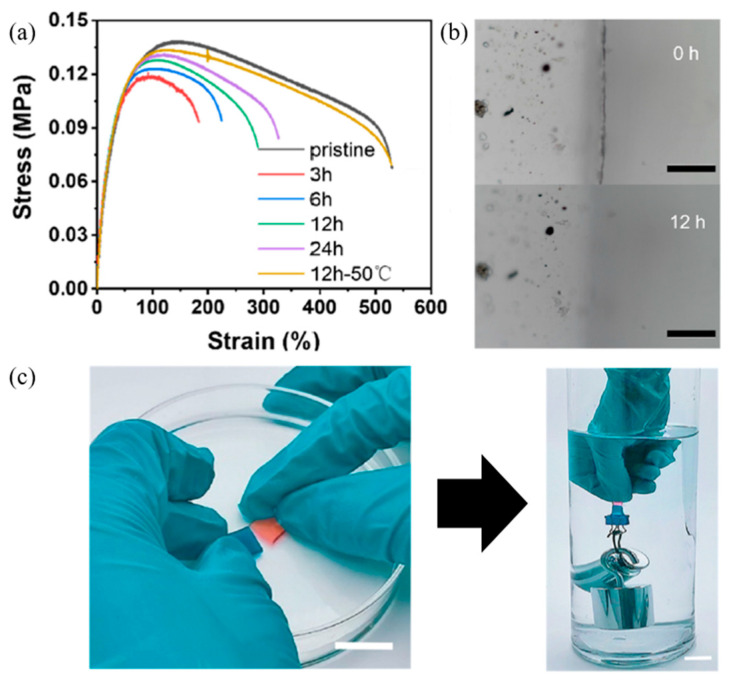
The experimental part of the self-healing process of fluorinated elastomer; (**a**) stress–strain curves of pristine and healed samples with various healing times, (**b**) optical images of healed samples after healing time for 12 h at 25 °C underwater, and (**c**) experimental part of the healed sample using lifting a weight underwater [110]. Adapted with permission from [110]. Copyright 2020 American Chemical Society.

Interestingly, self-healing underwater was studied by Zhang et al. (2020) [110]. In this research, poly(vinylidene fluoride-co-hexafluoropropylene) called fluorinated elastomer (FE) was dissolved and mixed in ionic liquids, which obtained multiple ion–dipole interactions in the molecules. The results in Figure 21 reveal that the mechanical properties are completely restored to their original stage after healing at 50 °C for 12 h due to the hydrophobic ion–dipole interaction. Furthermore, the crack of the damaged surface disappears after a healing time of 12 h. Therefore, the self-healing efficiency depends on both healing time and temperature.

**Figure 22:**
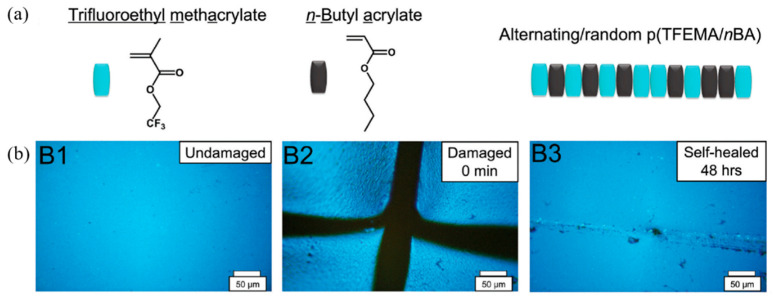
The self-healing process of fluorinated copolymers; (**a**) chemical structure of monomer in self-healing system; and (**b**) comparison of the optical images of undamaged, damaged, and healed samples [111]. Reprinted from [111], with permission from John Wiley and Sons.

Furthermore, Wang and Urban (2021) [111] presented self-healing of fluorinated copolymers. In the present research, trifluoroethyl methacrylate (TFEMA) and n-butyl acrylate (nBA) were copolymerized to obtain the random copolymer called p(TFEMA/nBA). The optical image in Figure 22 revealed the self-healing samples composed of a 50/50 TFEMA/nBA monomer molar ratio at 0 and 48 h. This result indicated that the combination of dipole–dipole interactions between C-F and C=O causes self-healing after 48 h.

The characteristics of dipole–dipole interactions in a self-healing system were investigated using FT-IR and summarized in Table 6.

## 7. Host–Guest Interaction of Polymers

Host–guest interaction is a type of non-covalent interaction that uses a principle similar to the lock and key principle, indicating specificity. The principle of the host–guest interaction relates to the receptor molecule acting as the host, and the ion acting as the guest. The host molecule must be specific in choosing to bind to the guest. Therefore, the lock and key concept is used to obtain the self-healing process.

**Figure 23:**
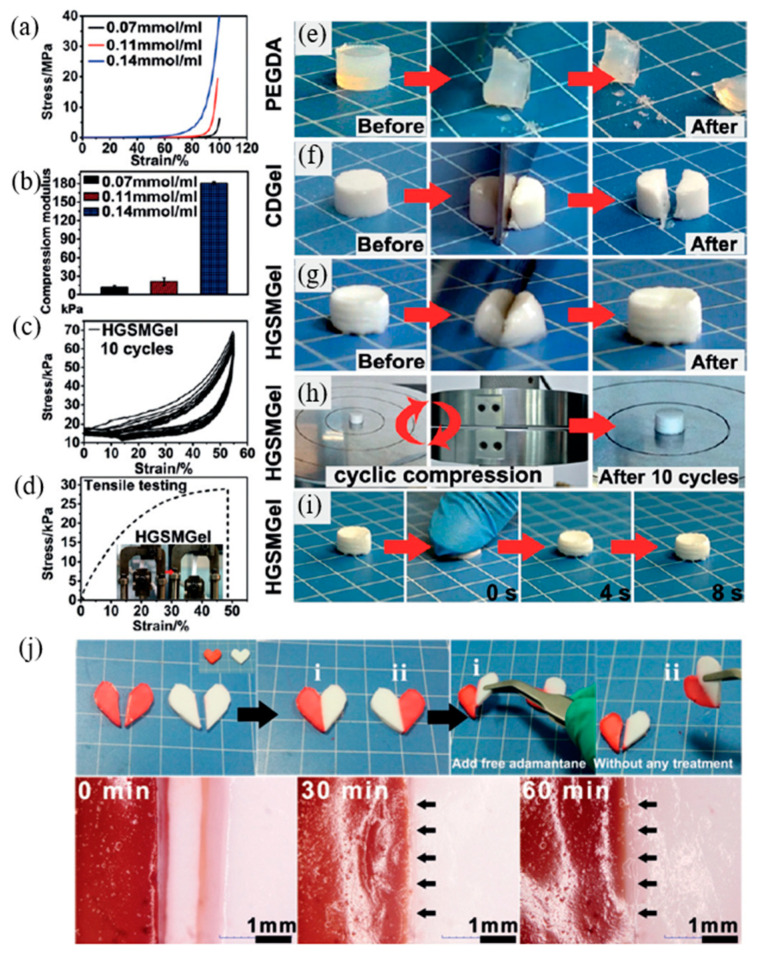
The mechanical properties of HGMS hydrogel in terms of (**a**) compression curves of hydrogel with various HGSM contents, (**b**) compression modulus of hydrogel with various HGMS contents, (**c**) cyclic compression test curves, and (**d**) stress–strain curve of HGSM hydrogel. The self-healing process of host–guest supramolecular hydrogels; (**e**–**g**) photographs of conventional hydrogel compared to HGSM hydrogel, (**h**,**i**) shape recovery of hydrogel after cyclic compression and compression by squashed, and (**j**) healing process and micrographs of healed samples [114]. Adapted from [114], with permission from John Wiley and Sons.

Wang et al. (2018) [114] studied rapid self-healing using the host–guest interaction in hydrogel. In this research, host–guest recognition was formed between a host of poly(isocyanatoethyl acrylate modified b-cyclodextrin) and a guest of 2-(2-(2-(2-(adamantyl-1-oxy)ethoxy)ethoxy)ethoxy)ethanol acrylate to obtain the host–guest supramolecular (HGSM) hydrogel. Then, HGSM hydrogel was crosslinked under UV-irradiated polymerization to obtain the covalent bonds in the hydrogel. The mechanical properties of the HGSM hydrogel (Figure 23) were presented in terms of tensile, compression, and cyclic compression testing. The result found that the stress–strain curves exhibit high strength of HGSM hydrogel. In terms of compressive modulus, the modulus was increased with concentration due to the higher crosslinking density. Then, the large hysteresis loops in the loading–unloading cycle of HGSM hydrogel represented its dissipated energy effectively. Moreover, the stretching length of HGSM hydrogel reached 48% and exhibited elasticity in this stretched state. Furthermore, the microscopy images revealed the self-healing process of the HGSM hydrogel for almost 60 min without any healable agent.

**Figure 24:**
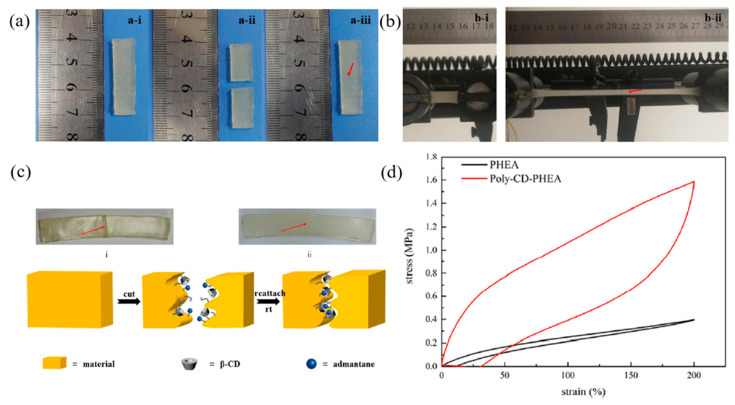
The self-healing of polycyclodextrin elastomer system with host–guest interaction; (**a**) photographs of healing process before and after healing time for 24 h, (**b**) photographs of healed sample under tension, (**c**) typical mechanism for self-healing materials, and (**d**) tensile loading-unloading curves of the toughening elastomers [115]. Adapted with permission from [115]. Copyright 2019 American Chemical Society.

The self-healing of toughening elastomers based on polycyclodextrin (poly-CDs) and methacryl-1-adamantane ethylene glycol diester (HEMA-Ad) was studied by Hou et al. (2019) [115]. The host–guest interaction was used for a healable process due to it being stable in moisture conditions and the fact that it was not affected by surface aging. Then, the poly-CDs and HEMA-Ad were multi-functional hosts and guest molecules, respectively. From a mechanical properties point of view, the hysteresis loop indicated the energy dissipation of materials. Figure 24 show that the toughening elastomers exhibit the energy dissipation more effectively compared to the poly(2-hydroxyethyl acrylate)-co-poly(methacryl-1-adamantane ethylene glycol diester) or PHEA during deformation because the synergistic effect of the host–guest interaction, representing the micro-network of poly-CD, and hydrogen bonding between polymer chains. Furthermore, the toughening elastomers revealed high strength, extensibility, and autonomously self-healing under ambient conditions [115].

**Figure 25:**
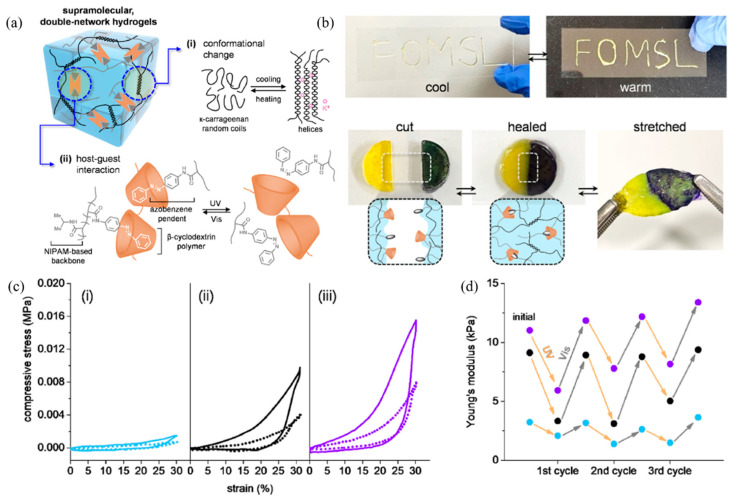
The self-healing of the supramolecular network in hydrogel; (**a**) the combination of reversible chains and host–guest interaction to obtain supramolecular networks, (**b**) demonstration of self-healing process, (**c**) compressive stress–strain curves of (i) ncSNH, (ii) ncDNH, and (iii) ncDNH at 36.5 °C before and after UV irradiation (solid and dotted), and (**d**) Young’s modulus changing the sample at various cycles of UV and visible light irradiation [116]. Adapted with permission from [116]. Copyright 2021 American Chemical Society.

Furthermore, Park et al. (2021) [116] designed and studied the supramolecular double-network in hydrogels containing reversible, non-covalent interactions. The supramolecular network in the hydrogel was also self-healable (Figure 25). In terms of the mechanical properties, the compressive stress–stain curve between the single-network hydrogel (ncSNH) and double-network hydrogel (ncDNH) were compared during a loading–unloading cycle. The results found that the Young’s modulus of ncDNH (9.3 ± 0.1 kPa) is higher than that of ncSNH (3.2 ± 0.1 kPa) due to the combination network in molecules. Interestingly, the hydrogel was more stiffed with an increase in temperature to 36.5 °C, which caused the additional formation of physical and interchain interactions together. Therefore, the increase in mechanical properties of self-healing material depends on the formation of network interactions in the molecules.

The characteristics of the host–guest interaction in the self-healing polymer were investigated using FT-IR and ^1^H-NMR, which are summarized in Table 7.

## 8. Conclusions and Perspective

Healable supramolecular polymers from the non-covalent interactions are an emerging innovation which can be designed for new applications in polymer technology. This type of supramolecular polymer can enhance mechanical properties in terms of reversible interactions for functional polymer products. Such self-healing phenomena may also be found in different types of non-covalent interaction, for example, metal–ligand coordination, hydrogen bonding, π–π interaction, electrostatic interaction, dipole–dipole interaction, and host–guest interaction. The challenge of performance is the control of the molecular structure; this relates to the program on the non-covalent interaction of supramolecular molecules. Thus, it is very festinated to better understand the relationship of method–structure–property for tailor-made supramolecular polymers. This relationship involves not only the chemical functionalized polymer structure based on the preparation method but also the mechanical properties of the self-healing phenomenon and the physical thermodynamics of both entropy and enthalpy changes.

A collection of approaches has been proposed by researchers to develop healable supramolecular polymers, summarized in Table 8. The data present self-healing systems using various non-covalent interactions, which can be obtained from chain movement to produce polymer matrix repairing. Interestingly, some of the systems can rapidly repair or self-heal, such as the hydrogen bonding of oxidized natural rubber/sodium alginate system and the π–π interaction of the polyimide/pyrenyl system. Furthermore, the results revealed that some self-healing processes could be easily obtained at room temperature. Therefore, we can adjust and apply the self-healing process with raw materials to produce good efficiency in terms of mechanical properties, energy dissipation, energy-saving, and cost.

From a human wound healing point of view, the self-healing mechanism from the non-covalent interaction may be applied for wound healing of humans in the future due to the intermolecular forces in the human body such as protein–protein, lipid–lipid, and hydrophobic interaction, etc. Furthermore, in terms of polymer utilization for self-healing products, this knowledge can be applied and developed to increase the lifetime of products, causing rapid healing, the reduction of accidents, and reduced maintenance costs of products such as surgical gloves, wound dressing, drug delivery materials, or even aircraft tires. Therefore, the future evolution of technologies is possible to apply this idea of molecular recognition, self-healing, and supramolecular force for non-covalent material utilization.

## Figures and Tables

**Table 1:** Market revenue of self-healing materials in the USA from 2020 to 2025 (in USD millions) [72].

Type of Product	Market Revenue (in USD Millions)					
	2020	2021	2022	2023	2024	2025
Fiber-reinforced composites	53.50	62.70	73.57	81.93	92.80	101.99
Concrete	53.50	64.37	73.57	82.76	91.96	99.48
Polymers	39.29	56.85	63.54	74.40	81.09	89.45
Coatings	46.82	43.47	52.67	59.36	67.72	75.24
Ceramic	28.42	35.11	39.29	45.98	50.16	54.34
Asphalt	19.65	23.83	28.01	33.02	38.87	43.89
Metals	25.92	30.93	35.11	36.78	40.13	43.47

**Table 2:** Overview of characterization methods for metal–ligand coordination.

Metal	Ligand	Characterization Methods			Ref.
		FT-IR	Raman	UV/VIS	
Fe^3+^	Catechol	-	512–627 cm^−1^ (catechol–iron bond vibrations)	-	[85]
Fe^3+^	Dopa	-	500–650 cm^−1^ (chelation of the Fe^3+^ by the oxygen of catechol)	-	[87]
Fe^3+^	Dopa	-	500–600 cm^−1^ (coordination between DOPA and Fe^3+^)	550 nm (coordination is enhanced between Fe^3+^ and catechol groups)	[90]
Zn^2+^	Acrylonitrile	2280 cm^−1^ (restricted −CN in Zn^2+^−CN coordination)	-	-	[92]
Zn^2+^	Terpyridine	-	-	392 nm (Zn^2+^-terpyridine coordination complex)	[93]
Ln^3+^	Terpyridine	1587, 1571, and 1562 cm^−1^ (C=N of terpyridine, related to complexation of terpyridine and Ln^3+^)	-	292 nm (π–π* transition of pyridine ring) and red-shifted to 324 nm (after the Ln^3+^ addition)	[94]

**Table 3:** Overview of characterization methods for H-bonding.

Materials	Characterization Methods		Ref.
	FT-IR	^1^H-NMR	
Oxidized natural rubber (oNR), Sodium alginate (SA)	3291 cm^−1^ is shifted to 3272 cm^−1^ (hydroxyl group) 1038 cm^−1^ is shifted to 1029 cm^−1^ (C-O stretching vibration of SA)	-	[76]
PVA, Dopamine	3306 cm^−1^ (broad and strong of OH stretching)	-	[95]
α,ω-Aminopropyl-terminated poly (dimethylsiloxane) (A-PDMS), Ethylene carbonate (EC)	1540 cm^−1^ (N−H bending) 1415 cm^−1^ (C−N stretching) 3420 cm^−1^ (broad and strong absorption bands of O−H)	7.6 ppm (N−H signals) 3.5 ppm (hydroxyl resonance)	[96]
Epoxidized natural rubber (ENR), Carboxymethyl chitosan (CMCS)	3425 cm^−1^ is shifted to 3388 cm^−1^ (hydroxyl group)	-	[97]
Aminopropyl-terminated polydimethylsiloxane (PDMS), 4,4′-methylenebis-(cyclohexyl isocyanate) (HMDI), 1,1′-Thiocarbonyldiimidazole (TCDI), Isophthalaldehyde (IPAL)	3340–3310 cm^−1^ (N−H stretching) 1700 cm^−1^ (C−O vibrations) 1500 cm^−1^ (Amide II band)	-	[98]

**Table 4:** Overview of characterization methods for π–π interaction.

Materials	Characterization Methods		Ref.
	FT-IR	UV/VIS	
Polyurethane, Polyimide	-	Broad absorption at 525 nm (π–π* charge-transfer transition between the electron-rich pyrenyl and electron-poor diimide residues)	[102]
Perylene terminated polymer, Poly(diimide)	-	Broad absorption band at 611 nm (blended solutions of perylene terminated polymer /chain-folding polydiimide)	[103]
Polystyrene (PS), Graphene	694, 749, 1386, and 1447 cm^−1^ (benzene ring of the PS segments), 2917 and 3020 cm^−1^ (methylene groups)	269.8 nm (ring currents in graphene and PS)	[104]
Polystyrene (PS), 6,13-bis((triisopropy-lsilyl)ethynyl) (TIPS)-pentacene	-	698 nm (TIPS-pentacene phase)	[105]

**Table 5:** Overview of characterization methods for electrostatic interaction.

Materials	Characterization Methods			Ref.
	FT-IR	UV/VIS	^1^H-NMR	
Poly (acrylic acid) (PAA), Poly(ethylene oxide) (PEO), oly(ethylenimine) (bPEI)	1550 cm^−1^ (COO− groups of PAA)	-	-	[106]
Acrylamide and acrylic acid copolymer (PAM), Alginate-modified dopamine (Alg-DA), Aluminum ions (Al^3+^)	2893 cm^−1^ (Stretching of C−H from −N+ (CH_3_)_2_−) 1215–1280 cm^−1^ (C−N stretching)	280 nm (successful synthesis of Alg-DA)	6.5–7.0 ppm (DA was successfully introduced to the alginate structure)	[107]
Polyaniline (PANI), Hydrophobic association poly(acrylic acid) (HAPAA)	1483 and 1561 cm^−1^ (stretching vibrations of the benzenoid and quinoid ring of PANI)	-	-	[108]

**Table 6:** Overview of characterization methods for dipole–dipole interaction.

Materials	Characterization Methods	Ref.
	FT-IR	
1-Methylimidazole, Bis(2-bromoethyl) ether, EMITFSI, Lithium bis(trifluoromethanesulphonyl) imide (LiTFSI)	1346 cm^−1^ shifted to 1351 cm^−1^ (S=O stretching band), 1051 cm^−1^ shifted to 1055 cm^−1^ (N−S stretching band)	[110]
1,1,3,3-tetramethylurea (TMU), acetonitrile (CH_3_CN) and carbon tetrachloride (CCl4)	1653 cm^−1^ (TMU and CD_3_CN interaction and slight shift of the carbonyl band of TMU around)	[112]
Amphiphilic compounds (N+C10-Azo-Gly-OC2Rfn: NAGFn)	1148.9 cm^−1^ (the position of the νs(CF_2_) band)	[113]

**Table 7:** Overview of characterization methods for host–guest interaction.

Materials	Characterization Methods		Ref.
	FT-IR	^1^H-NMR	
Isocyanatoethyl acrylate modified b-cyclodextrin (b-CD-AOI2), 2-(2-(2-(2-(adamantyl-1-oxy)ethoxy)ethoxy)ethoxy)ethanol acrylate (A-TEG-Ad)	1534 and 1635 cm^−1^ (stretching vibrations of the secondary amides)	4.9 ppm (signal integral area ratio between C_1_-H of b-CD) 5.8–6.4 ppm (double bond, −CH=CH_2_)	[114]
Copolymerization of epichlorohydrin (EP) and β-cyclodextrin (CD)	-	1.6–2.2 ppm (protons of Ad functional group), 3.0–4.0 ppm (internal proton of β-CD)	[115]
Azo-acrylamide, β-cyclodextrin polymer (bCDP)	-	3.3–4 ppm (protons located in the cavity of the cyclodextrin units in bCDP), 6–8 ppm (The protons on sp^2^ carbons of azo-acrylamide)	[116]

**Table 8:** Summary of self-healing systems.

No.	Materials	Self-Healing Character	Preparation Method	Self-Healing Condition	Self-Healing Efficiency	Ref.
1	ENR, Dopamine, Fe^3+^	M-L coordination	Mixing and compression molding	50 °C, 12 h	Elongation: 95.2 ± 15.9%, Tensile strength: 86.1 ± 5.2%.	[88]
2	Silicone rubber, Dopa, Fe^3+^	M-L coordination	Mixing and pouring into a Petri dish	120 °C, 84 h and pH = 9 underwater	Elongation: 84 ± 2%	[89]
3	ENR, Pyridine, Fe^3+^	M-L coordination	Mixing and pouring and spray coating	60 °C, 8 h	Tensile strength: 87%	[90]
4	PVA, Succinic anhydride, Dopamine	H-bonding	Melt processing	25 °C, 24 h 25 °C, 48 h	Tensile strength: 10.87% Tensile strength: 17.39%	[95]
5	Silicone rubber, Ethylene carbonate	H-bonding	Mixing and pouring into a mold	80 °C, 24 h	Tensile strength: 88.5%	[96]
6	ENR, Carboxymethyl chitosan	H-bonding	Solution mixing and pouring into a mold	RT, 12 h	Tensile strength: 90%	[97]
7	Oxidized NR, Sodium alginate	H-bonding	Mixing and pouring into a mold	RT, 5 min	Elongation: 94%	[76]
8	Silicone elastomer, Thiourea	H-bonding	Mixing and pouring into a mold	RT, 24 h	Tensile strength: 94.9%	[98]
9	Polyimide, Pyrenyl	π–π interaction	Mixing	87 °C, 5 min	Tensile modulus: 100%	[101,102]
10	Poly(diimide), Perylene	π–π interaction	Mixing	75 °C, 14 min or 125 °C, 30 min	Shear modulus: 100%	[103]
11	Poly(ethylenimine), Poly(acrylic acid), Poly(ethylene oxide)	Electrostatic interactions and H-bonding	Dropwise mixing and compression molding	RT, 48 h	Elongation: 96%	[106]
12	DA, Al^3+^	Electrostatic interactions, M-L coordination, H-bonding	Mixing	RT, 24 h	Tensile strength: 91.83%	[107]
13	PA, PANI, HAPAA	Electrostatic interactions, H-bonding	Pre-infiltration method	RT, 72 h	Tensile strength: 70% Electrical: 92%	[108]
14	Fluorinated elastomer, Dibutyl phthalate	Dipole–dipole interactions	Mixing	RT, 24 h and underwater	Elongation: 30.61 ± 3.97%	[109]
15	Fluorinated elastomer, Ionic liquids	Dipole–dipole interactions	Mixing	50 °C, 12 h	Elongation: 100%	[110]
16	Poly(isocyanatoethyl acrylate modified b-cyclodextrin), Acrylate compound	Host–guest interaction	Mixing	RT, 60 min	Tensile strength: 60%	[114]
17	Polycyclodextrin, Methacryl-1-adamantane ethylene glycol diester	Host–guest interaction	Mixing	RT, 24 h and moisture conditions	Tensile strength: 75%	[115]

## Data Availability

No new data was generated for this review paper.
